# Nitric oxide-based regulation of metabolism: Hints from TRAP1 and SIRT3 crosstalk

**DOI:** 10.3389/fmolb.2022.942729

**Published:** 2022-07-26

**Authors:** Fiorella Faienza, Andrea Rasola, Giuseppe Filomeni

**Affiliations:** ^1^ Department of Biology, University of Rome Tor Vergata, Rome, Italy; ^2^ Department of Biomedical Sciences, University of Padova, Padova, Italy; ^3^ Redox Biology, Danish Cancer Society Research Center, Copenhagen, Denmark; ^4^ Center for Healthy Aging, University of Copenhagen, Copenhagen, Denmark

**Keywords:** nitric oxide, metabolism, mitochondria, SDH, nitrosylation

## Nitric oxide in metabolism regulation

Nitric oxide (NO) is a gaseous signaling molecule able to modify protein structure and activity (Rizza and Filomeni, 2017). The addition of the NO moiety to a cysteine thiol is called *S*-nitrosylation, which is establishing as one of the major NO-induced protein posttranslational modifications involved in cell signaling (Fernando et al., 2019). In the presence of oxygen—and reactive oxygen species (ROS), mostly superoxide (O_2_^-^)—NO can also give rise to other biologically active compounds, such as peroxynitrite (ONOO^−^) (Fernando et al., 2019). Peroxynitrite is highly reactive and commonly recognized being detrimental to macromolecules, as it can cause irreversible modifications to lipids (peroxidation) and proteins (tyrosine nitration) (Radi, 2018).

Nitric oxide is endogenously generated by nitric oxide synthases (NOSs), a family of three isozymes (neuronal, endothelial, and inducible) whose subcellular localization and activation influence NO target selectivity (Benhar et al., 2009). NO and NO-derived species (e.g., N_2_O_3_, NO^+^) can directly react with thiol groups in proteins or in sulfhydryl-containing low molecular weight molecules, such as glutathione, free cysteine, and Coenzyme A (CoA). Otherwise, S-nitroso (SNO) groups can be generated *via trans*nitrosylation, the transfer of an NO moiety between thiols and S-nitrosothiols (thiol/nitrosothiol exchange) (Nakamura et al., 2021). This mechanism is also the basis of protein denitrosylation, which is the process required to remove NO from S-nitrosothiols and regenerate protein thiol pools. Three are the denitrosylases so far characterized: i) *S*-nitrosoglutathione reductase (GSNOR); ii) *S*-nitroso-CoA reductase (ScoR) and iii) thioredoxin reductase (TrxR). All these enzymes do not directly react with *S*-nitrosylated proteins, but specifically recognize, and remove, the NO moiety from small S-nitrosothiols (S-nitrosoglutathione, *S*-nitroso-CoA and thioredoxin, respectively) generated by *trans*nitrosylation with S-nitrosylated proteins (Rizza and Filomeni, 2017).

*S*-nitrosylation usually acts as an inhibitory mechanism of key metabolic enzymes in order to adapt metabolism to cell’s needs (Brown, 2007). Nitric oxide inhibits all electron transport chain (ETC) complexes (Poderoso et al., 2019). Cytochrome *c* oxidase (CcOX), the complex IV of ETC (Rich, 2017), represents the best-characterized mitochondrial target of NO. At low levels, NO inhibits complex IV competing with O_2_ for the binding to the active site, whereas, at high concentrations, the inhibition occurs *via S*-nitrosylation (Cleeter et al., 1994). The NO-mediated regulation of CcOX activity is closely related to the oxygen availability and finely tunes cellular respiration, as it controls the balance between O_2_ cellular uptake and consumption (Poderoso et al., 2019). As a side-effect of NO-mediated inhibition of mitochondrial complexes, ROS are produced, this leading to the production of peroxynitrite that further enhances NO inhibition on ETC complexes in a positive feedback loop (Brown, 2007).

NO also inhibits several tricarboxylic acid (TCA) cycle enzymes, such as aconitase, α-ketoglutarate dehydrogenase and succinate dehydrogenase (SDH) (Prime et al., 2009), as well as enzymes involved in fatty acid oxidation (FAO) (Piantadosi, 2012) and branched-chain amino acid metabolism (Coles et al., 2009). Likewise, pyruvate kinase M2 is inhibited by *S*-nitrosylation, redirecting glucose towards the pentose phosphate pathway (Lowenstein, 2019). Nitric oxide can also tune metabolism by targeting the chaperone tumor necrosis factor receptor-associated protein 1 (TRAP1) and the deacetylase sirtuin 3 (SIRT3) (Rizza et al., 2016; Kalous et al., 2021), which have been recently proposed to be mutually regulated in the mitochondria (Park et al., 2019).

## Tumor necrosis factor receptor-associated protein 1

### Tumor necrosis factor receptor-associated protein 1 in metabolism

TRAP1 is a mitochondrial chaperone belonging to heat shock protein 90 family (Hoter et al., 2018). It is considered a key molecule for the maintenance of mitochondrial homeostasis given its ability to: i) promote metabolism rewiring by sustaining a “Warburg-like”, aerobic glycolysis phenotype; ii) downregulate ROS production, iii) protect from stress-induced cell death (Hoter et al., 2018). TRAP1 works as a dimer in which each monomer is composed of three domains: 1) the N-terminal domain, responsible for ATP binding; 2) the *middle* domain, which participates in ATP binding site shaping and client allocation; 3) the C-terminal domain, indispensable for dimerization (Serapian et al., 2021). Interestingly, it has been recently demonstrated that TRAP1 is also able to generate tetramers (i.e., dimers of dimers) in association with changes in oxidative phosphorylation (OXPHOS) rate (Joshi et al., 2020; Liu et al., 2020).

Only few TRAP1 interactors, called clients, have been characterized to date (Sanchez-Martin et al., 2020b), even if emerging data indicate that TRAP1 can bind multiple mitochondrial components of bioenergetic circuits (Joshi et al., 2020; Cannino et al., 2022). Among the best defined TRAP1 clients is SDH, a key enzyme placed at the intersection of OXPHOS and TCA cycle. TRAP1 downregulates SDH activity (Sciacovelli et al., 2013), an effect reinforced by TRAP1 interaction with the mitochondrial fraction of the extracellular signal-regulated kinase (ERK) (Masgras et al., 2017a). Consequently, besides dampening OXPHOS rate, TRAP1-mediated inhibition of SDH activity results in succinate accumulation (Faienza et al., 2020b), an effect particularly relevant in cancer models characterized by hyperactivation of Ras/ERK signalling (Masgras et al., 2017b). Indeed, succinate is an oncometabolite that concurs to promote tumorigenesis by affecting the activity of α-ketoglutarate dependent hydroxylases (Islam et al., 2018). Among this huge class of enzymes, it is worth to mention: i) 5-methylcytosine hydroxylases and JmjC domain containing lysine demethylases, which broadly affect epigenetic regulation, and ii) prolyl hydroxylases, which leads to stabilization of the hypoxia inducible factor 1α (HIF-1α) and activation of a HIF1-mediated (pseudo)hypoxic transcription program, condition favoring neoplastic progression even under normoxia (Sanchez-Martin et al., 2020b; Masgras et al., 2021). TRAP1 is transcriptionally activated by HIF1, in a feed-forward loop between HIF1 and TRAP1, which sustains metabolic adaptations in cancer, as well as during embryonic development (Laquatra et al., 2021) and oxidative damage associated to ischemic conditions (Masgras et al., 2017b). The inhibitory interaction between TRAP1 and the mitochondrial fraction of the c-Src kinase, a CcOX activator (Yoshida et al., 2013), could also contribute to OXPHOS inhibition, in line with the antioxidant effect exerted by TRAP1 (Guzzo et al., 2014).

In agreement with its pro-neoplastic role, TRAP1 also inhibits cell death. Recent findings suggest that this could rely on TRAP1 interaction with F-ATP synthase: the OXPHOS complex that generates ATP and acts as the permeability transition pore (PTP) (Urbani et al., 2019), a mitochondrial channel whose opening commits cells to death (Masgras et al., 2021). Indeed, TRAP1 interacts with the F-ATP synthase subunit OSCP, blocking the PTP channel activity and outcompeting the PTP-activating interaction between OSCP and the prolyl isomerase cyclophilin D (Cannino et al., 2022).

### Tumor necrosis factor receptor-associated protein 1 regulation by nitric oxide

TRAP1 is a target of *S*-nitrosylation, which affects protein stability and activity (Faienza et al., 2020b). This is particularly relevant in a GSNOR-deficient model of hepatocellular carcinoma (HCC) where GSNOR loss increases SDH activity and, in turn, confers high sensitivity to SDH-targeting drugs (Rizza et al., 2016). This mitochondrial alteration represents the *Achilles’ heel* of GSNOR-deficient HCC cells and is associated with TRAP1 *S*-nitrosylation at Cys501, which causes TRAP1 destabilization and proteasomal degradation (Rizza et al., 2016). *S*-nitrosylation of Cys501 also reduces TRAP1 ATPase activity *via* an allosteric mechanism, and cells expressing the non-nitrosylable TRAP1 mutant are less sensitive to apoptosis induction (Faienza et al., 2020a). This suggests that Cys501 nitrosylation inhibits TRAP1 ATPase activity, destabilizes its structure and directs TRAP1 to proteasomal degradation (Faienza et al., 2020b) or, as proposed (Elnatan et al., 2017), promotes a TRAP1 holdase activity that shields clients exposed to stress conditions in an ATP-independent way.

## Sirtuin 3

### Sirtuin 3 in metabolism

Sirtuins (SIRTs) are NAD^+^-dependent deacetylases with a catalytic site where four cysteine residues coordinate a Zinc ion (Zn^2+^-tetrathiolate) (Kalous et al., 2021). Since sirtuins need NAD^+^ to work, they are both sensors and regulators of the NAD^+^/NADH ratio, which affects metabolism and the redox status of cells (Elkhwanky and Hakkola, 2017). Seven sirtuins have been described in mammals, three of which (SIRT3, SIRT4, and SIRT5) are localized in mitochondria. SIRT3 is the main mitochondrial deacetylase, whereas SIRT4 is involved in ADP-ribosylation and SIRT5 in desuccinylation, demalonylation, and deglutarylation reactions (Singh et al., 2017a).

SIRT3 has been implicated in virtually all mitochondrial metabolic pathways (Rardin et al., 2013), as it controls the activity of a variety of mitochondrial enzymes involved in redox and bioenergetic homeostasis by regulating their acetylation status (Kalous et al., 2021). Mitochondrial translocation of SIRT3 is increased in response to several stress stimuli (Singh et al., 2017a).

The first mitochondrial proteins identified as SIRT3 targets were complex I and II of the ETC (Bong-Hyun et al., 2008), but further studies suggested that all ETC complexes can be regulated by SIRT3 (Bell et al., 2011). Numerous observations extended the number of SIRT3 substrates, which currently includes α-ketoglutarate dehydrogenase and malate dehydrogenase 2 Rardin al. (2013), whereas isocitrate dehydrogenase 2 and superoxide dismutase 2 (SOD2) are activated by SIRT3-dependent deacetylation (Zullo et al., 2022), leading to an increase in NADPH levels and in the antioxidant response (Yu et al., 2012). In further agreement with the antioxidant function of SIRT3, it is worth noting that its deacetylating activity is required for Forkhead box O3a-mediated transcription of both superoxide dismutase 2 and catalase (Singh et al., 2017a).

A large body of literature defines SIRT3 as a general booster of mitochondrial metabolism. Enzymes belonging to the urea cycle, FAO (Hirschey et al., 2010) and ketogenesis (Shimazu et al., 2010) are activated by SIRT3 deacetylation to keep metabolism running. Phenotypes observed in SIRT3 KO (*Sir3* ^−/−^) mice support this general idea, showing: i) a general decrease of cellular respiration (Bong-Hyun et al., 2008; Singh et al., 2017a); ii) liver steatosis associated with increased levels of triglycerides and FAO intermediates (Hirschey et al., 2010; Rardin et al., 2013); iii) alterations in ketone body production (Elkhwanky and Hakkola, 2017).

### Sirtuin 3 regulation by nitric oxide

Zn^2+^-tetrathiolate is a target of several oxidative post-translational modifications (Kalous et al., 2021). Among these, *S*-nitrosylation has been proposed to have a prominent role, and the S-nitrosylated form of Zn^2+^-tetrathiolate has a general inhibitory effect on sirtuins. However, in mitochondria, NO generated by NOS1 *activates* SIRT3, resulting in deacetylation-dependent SOD2 activation and down-regulation of ROS levels (Wang et al., 2019). Vice versa, massive NO production has been reported to inactivate SIRT3 *via* tyrosine nitration (Pérez et al., 2018). This argues for a Janus-faced (physio-pathological) effect for NO, depending on the fluxes applied, which is consistent with the crucial role of SIRT3 in the antioxidant defense of the cell (Kalous et al., 2020).

## Tumor necrosis factor receptor-associated protein 1 and sirtuin 3 crosstalk

### Tumor necrosis factor receptor-associated protein 1 and sirtuin 3 in cancer

An interaction between TRAP1 and SIRT3 has been reported in glioma stem cells (GSC) (Park et al., 2019), where the two proteins reciprocally sustain their activities: SIRT3-mediated deacetylation maintains TRAP1 chaperone activity, which in turn stabilizes SIRT3. Several OXPHOS components associate with TRAP1/SIRT3 complex, resulting in a high level of respiration matched by low ROS. However, in line with the metabolic plasticity that characterizes neoplastic cells, it is possible to envisage other metabolic effects of TRAP1 and SIRT3. For instance, in malignant cells related to the tumor-predisposing syndrome neurofibromatosis type 1 (NF1), the hyperactivation of Ras/ERK signaling, which occurs downstream to loss of neurofibromin, dampens the expression and activity of complex I, lowering both respiration and intracellular NAD^+^ levels. As a result, SIRT3 activity is reduced, supporting the hypothesis that SIRT3 has anti-neoplastic effects that synergize with TRAP1 inhibition (Masgras et al., 2022). Indeed, both SIRT3 induction and TRAP1 inhibition enhance SDH enzymatic activity to a similar extent and without any additive effect. Coherently, the allosteric TRAP1 inhibitor honokiol bis-dichloroacetate increases SDH activity at the same level of SIRT3 overexpression in NF1-related malignant peripheral nerve sheath tumor cells (Sanchez-Martin et al., 2020a). Even if these results are in contrast with the SIRT3-dependent activation of TRAP1 observed in GSC (Park et al., 2019), they provide a glimpse into a fascinating scenario in which SIRT3 and TRAP1 can harmonize the bioenergetic features of tumor cells with their needs and supplies, and provide a regulatory backbone that dynamically interacts with key metabolic components.

### Nitric oxide in tumor necrosis factor receptor-associated protein 1 and Sirtuin 3 crosstalk

**FIGURE 1:**
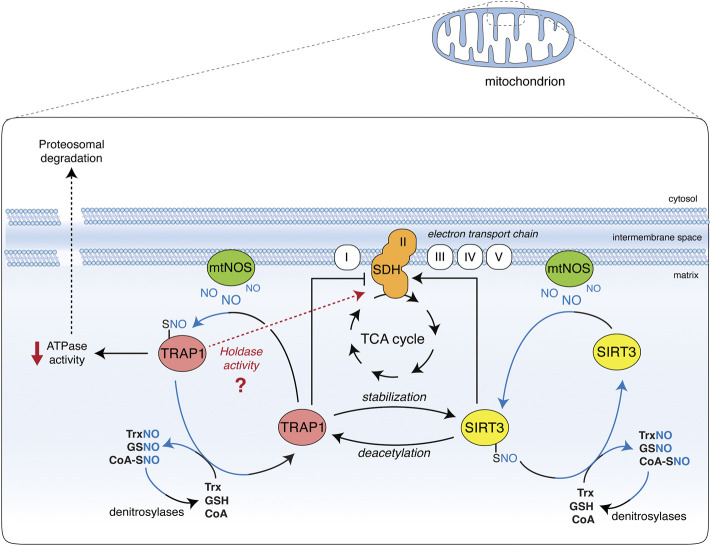
**Effect of TRAP1 and SIRT3 S-nitrosylation on SDH**. Nitric oxide (NO) is generated in the mitochondria by the mitochondrial portion of NOS2 (mtNOS). Besides nitration-induced irreversible damage (not shown), it causes the S-nitrosylation of TRAP1 and SIRT3 (TRAP1-SNO and SIRT3-SNO), which are in equilibrium with low molecular weight nitrosothiols (molecules and small proteins, such as GSNO, SNO-CoA, and TrxNO), the levels of which are controlled by denitrosylases (GSNOR, SCoR, and TrxR, respectively). S-nitrosylation increases SIRT3 activity, hence promoting succinate dehydrogenase (SDH) deacetylation and activation. SIRT3 has also been reported to deacetylate and activate TRAP1, which is crucial for SIRT3 protein stability. On the other hand, S-nitrosylation inhibits TRAP1 ATPase activity and promotes its degradation *via* the proteasome. Being TRAP1 a negative regulator of SDH, SDH activity is consequently increased. TRAP1 *S*-nitrosylation may also be responsible for a shift towards a holdase-like function of the protein (as recently proposed). This additional ability of TRAP1 could alternatively affect SDH stability and activity, and may help reconcile discrepancies between different studies on the effects of SIRT3 on SDH: if it takes place or not *via* TRAP1 inhibition or induction.

Nitric oxide allosterically inhibits TRAP1 ATPase activity, perturbing long-range structural communications and conformational changes (Faienza et al., 2020a). It also stimulates SIRT3 deacetylates activity (Wang et al., 2019) in a way resembling the effects of HDCA on both proteins (Sanchez-Martin et al., 2020a), and suggesting a common route that converges to regulate SDH activity. In conditions of NO toxicity (e.g., at very high concentrations), SDH is inhibited, most likely as a final effect of peroxynitrite-mediated protein damage and irreversible inactivation (Brown, 2007). However, in pathophysiological models of defective denitrosylation (e.g., in GSNOR-deficient systems), NO causes TRAP1 *S*-nitrosylation and degradation, which results in an increase in SDH activity (Rizza et al., 2016). This effect suggests that NO positively regulates SDH *via S*-nitrosylation through a dual mode, i.e., by inhibiting TRAP1 ATPase activity and activating SIRT3 (Figure 1). These opposite effects are mirrored by the dual role of NO in tumors, which finally results in driving metabolic adaptions of tumor cells. Notably, a switch between the chaperone and holdase-like activities of TRAP1—in which NO probably plays a role—may add a further level of complexity in the regulation of TRAP1/SIRT3 interaction (Figure 1).

## Conclusion

Although the role of NO in metabolism is well-established, most of the studies takes into account the direct inhibitory effect of NO on metabolic enzymes, and barely consider other layers of regulation. In this Opinion paper we have elaborated on, and proposed that NO-dependent regulation of metabolism could go beyond this direct effect and influence key mitochondrial regulators, i.e., TRAP1 and SIRT3, which are involved in cancer-associated increase of antioxidant response and metabolic rewiring (Singh et al., 2017a; Sanchez-Martin et al., 2020b). This is particularly relevant in neoplastic progression, where both TRAP1 and SIRT3 can simultaneously confer the ability to cope with oxidative stress and adapt to metabolic changes.

## Abbreviations

CoA, coenzyme A; CcOX, cytochrome c oxidase; ETC, electron transport chain; FAO, fatty acid oxidation; GSC, glioma stem cells; GSNOR, S-nitrosoglutathione reductase; HCC, hepatocellular carcinoma; HIF-1, hypoxia inducible factor 1; NO, nitric oxide; NF1, neurofibromatosis 1; OXPHOS, oxidative phosphorylation; PTP, permeability transition pore; ROS, reactive oxygen species; SDH, succinate dehydrogenase; SIRT, sirtuin; TCA, tricarboxylic acid; TRAP1, tumor necrosis factor receptor-associated protein 1.
